# “The COVID-19 pandemic and operational challenges, impacts, and lessons learned: a multi-methods study of U.S. prison systems”

**DOI:** 10.1186/s40352-023-00253-6

**Published:** 2023-12-05

**Authors:** Meghan A. Novisky, Jennifer Tostlebe, David Pyrooz, Jose Antonio Sanchez

**Affiliations:** 1 Department of Criminology and Sociology, Cleveland State University, 2121 Euclid Avenue, UR 205, Cleveland, OH 44115 USA; 2 School of Criminology and Criminal Justice, University of Nebraska Omaha, Omaha, NE USA; 3 Department of Sociology, University of Colorado Boulder, Boulder, CO USA

**Keywords:** Prisons, COVID-19, Infectious disease, Correctional staff, Health

## Abstract

**Background:**

The purpose of this study was to examine how the COVID-19 pandemic changed U.S. prison operations and influenced the daily work of prison staff.

**Methods:**

In collaboration with the National Institute of Corrections, we administered a survey to 31 state correctional agencies in April 2021 and conducted five focus groups with 62 correctional staff.

**Results:**

Using a framework of bounded rationality, we find that daily operations were strained, particularly in the areas of staffing, implementing public health policy efforts, and sustaining correctional programming. While prison systems and staff were under-prepared to respond to the pandemic, they attempted to address complex problems with the limited resources they had.

**Conclusions:**

Results underscore a need in corrections for prioritizing further developments and reviews of collaborative policies and practices for managing crisis situations. Seeking avenues for leveraging technological innovations to improve operations and facilitate enhanced communication are especially warranted. Finally, meaningful reductions in the prison population, changes in physical infrastructure, and expansions of hiring and retention initiatives are critical for positioning prisons to manage future emergencies.

## Introduction

The coronavirus 2019 (COVID-19)  pandemic presented unprecedented challenges to the functioning of core institutions in the United States and across the world. Beginning in March 2020, the norms and routines of society’s major institutions—the family, government, economy, education, and religion—shifted drastically in hopes of reducing the health-related impacts of the pandemic. However, with this shift came economic, societal, and organizational changes. Within the United States, various degrees of pandemic responses were observed (Hallas et al., 2020), including variation in the designation of industries, agencies, and occupations that provide services critical to societal operations (i.e., essential industries/workers; Center for Disease Control & Prevention, 2022; National Conference of State Legislatures, 2022). First responders, including those employed at correctional institutions, were designated as essential workers and tasked with swiftly deploying resources and policies to help facilitate public health efforts and mitigate the spread of COVID-19 (hereafter referred to as COVID). State and federal prisons were at the forefront of pandemic response efforts, as these institutions maintain custody of the bulk of incarcerated people. Together, these facilities house over 1.2 million individuals (Carson, 2022) and employ approximately 225,000 correctional officers (U.S. Bureau of Labor Statistics, 2021).

There was little doubt that prisons would be a vector of COVID transmission. Indeed, advocates and social scientists were sounding the alarm in early 2020 (Kinner et al., 2020; Oladeru et al., 2020). Given the large quantity of people incarcerated in prisons throughout the U.S., coupled with the environmental characteristics common to prison architecture and housing designations (e.g., overcrowding, restricted movement, inability to social distance, poor sanitation and ventilation, insufficient access to or poor quality health care), a virus like COVID was expected to wreak havoc on the health and wellbeing of those who work and live in correctional environments (e.g., Novisky et al., 2021; Pyrooz et al., 2020). Incarcerated individuals were also inevitably and uniquely vulnerable to COVID-19 infection and death, as the carceral population is filled with individuals at heightened risk for severe illness and co-morbidities at baseline (Akiyama et al., 2020; Kinner et al., 2020; Wildeman & Wang, 2017).

As anticipated, Departments of Correction (DOCs) experienced many COVID infections and deaths. As of July 3, 2023, 647,349 incarcerated people and 246,858 staff working in prisons have tested positive for COVID, while 2,933 prisoners and 292 staff have died due to the disease (COVID Prison Project, 2023). The authors of a recent scoping review found that COVID incidence and prevalence rates in carceral settings have consistently exceeded rates in the general population by three to five times (Puglisi et al., 2023). Further, this same study concluded that COVID hospitalization outcomes have been significantly worse among carceral populations relative to community populations (e.g., higher intubation rates, readmission rates, ICU stays) and mortality rates among incarcerated people and staff have exceeded community rates by two to four times. Over a three-year time period (i.e., 2017 to 2019), 9,599 individuals incarcerated in state prisons died from illness-related deaths (Carson, 2021a, 2021b). The majority of which included deaths due to cancer (*n* = 3,333) and heart disease (*n* = 3,194), followed by respiratory disease (*n* = 778). While COVID deaths do not exceed those from cancer and heart disease, it is ranked among the top three most deadly illness-related causes of death within correctional facilities.

Tasked with large scale pandemic response efforts early on, correctional facilities faced numerous challenges in their implementation of COVID mitigation efforts, including medical supply and staffing shortages, transient populations, and overcrowding (Williams et al., 2020). As with other sectors in society, there was also variation in what measures to implement (e.g., masking, modified co-payment policies, quarantine) and when (Herring & Sharma, 2021; Maner et al., 2021). Two studies documented early federal Bureau of Prisons and state DOC responses to COVID (see, Hummer, 2020 and Novisky et al., 2020, respectively). Additionally, a Bureau of Justice Statistics report was released about state and federal prison responses to the pandemic, but focused on summarizing descriptive data (e.g., number of infections, number of COVID tests) collected between March, 2020 and February, 2021 (Carson & Nadel, 2022).

Missing from the literature is an attempt to assess how and why U.S. prison operations changed in response to the COVID pandemic, from the perspective of correctional staff who were responsible for spearheading and implementing pandemic response efforts. This takes on added significance in light of early—and continued—calls to “establish best practices and facilitate public health functions” (Montoya-Barthelemy et al., 2020, p. 4; see also Puglisi et al., 2023) and to understand “how the pandemic has influenced the daily work of frontline prison staff” (Schultz & Ricciardelli, 2022, p. 2).

This study examines the pandemic's impact on prison operations. We do this by drawing on two sources of original data to provide a mixed-methods study on the COVID pandemic in U.S. prisons, including a survey administered to executive officials in state correctional agencies and focus groups with correctional personnel— ranging from line staff to wardens—from across the country. Using the framework of bounded rationality theory (Simon, 1953, 1956), we build upon prior research to extend the literature in three critical ways. First, we provide a comprehensive summary of custodial and staff population changes between January 2020 and January 2021 (approximately one year into the pandemic), strategies executed, and clinical management practices implemented to prevent the spread of COVID. Second, our data offer important theoretical context about how the pandemic influenced carceral policy. Finally, we expand what is known about working in carceral environments during an unprecedented period of crisis, which can help provide guidance to ongoing and future pandemic-related emergencies.

## Background

### The challenge of infectious disease in custodial settings

Priorities in correctional facilities routinely center around security concerns rather than the health and wellbeing of those who live and work in the facilities. The COVID pandemic drastically altered the carceral landscape by bringing discussions of health to the forefront in unprecedented ways. In early March 2020, the Centers for Disease Control and Prevention (CDC) released interim guidelines on the management of COVID in correctional facilities, which were organized into three sections with concrete strategies for prevention:

1. *operational preparedness:* for example, communication with incarcerated persons and community partners about COVID; review existing plans and revise for COVID; coordinate with local law enforcement and court officials; review personnel practices and identify duties that can be performed remotely; ensure sufficient stocks of hygiene, cleaning, protective personal equipment (PPE), and medical supplies;
2. *prevention of disease transmission:* for example, limit visitation, transfers, and in-person court appearances; provide cloth masks at no cost to incarcerated persons and launder them routinely; implementing social distancing strategies; perform verbal screening and temperature checks for all staff daily on entry; and
3. *clinical management:* for example, medical isolation and care of persons with COVID; quarantining close contacts; wearing PPE; cleaning and disinfecting areas; and contact tracing.^1^

Using this three-pronged approach, correctional leaders across the country mobilized to identify and implement strategies to reduce the impact of COVID in their facilities. Strategies included decreasing admissions to state DOCs; administering COVID tests to incarcerated people; suspending visitation and programming; using quarantine and lockdown procedures; suspending transfers between prisons and jails; providing face masks to incarcerated people and staff; and taking staff temperature checks prior to entering facilities (e.g., Carson & Nadel, 2022). Despite these targeted recommendations, correctional leaders and staff have faced many challenges in the course of their implementation.

One challenge to implementing the recommended CDC guidelines included the fact that prisons are a high-risk setting for the spread of COVID and other infectious diseases. For example, social distancing efforts had limited opportunities for success given the large—often overcapacity—population sizes managed by prisons and open bay/dormitory housing styles, which, by default, ensures incarcerated people and staff are in close proximity on a regular basis (Hawks et al., 2020). Additionally, prisons tend to house high concentrations of people with pre-existing chronic health issues (e.g., cancer, asthma, hypertension, diabetes, HIV, hepatitis) relative to the general population (Fahmy & Wallace, 2018), and an aging custodial population with extensive health needs (Carson & Sabol, 2016; Novisky, 2018; Williams et al., 2021). Finally, prison staff themselves are vectors of disease, as community COVID spread contributes significantly to COVID case rates in prisons (LeMasters et al., 2022) as staff enter and exit facilities frequently.

A second challenge included the rapidly changing “best practices” for controlling the virus within a high-risk setting. While the initial CDC guidelines appeared in early March 2020, *many* revisions were released (and still are being released) based on what was/is currently known about the transmission and severity of COVID. While community settings were also confronted with frequent changes in public health guidance, there were distinct challenges in managing correctional facilities, particularly in the absence of predictable expectations and consistency. Using in-depth interview data with twenty-one correctional officers in a Southern U.S. jail, Ferdik and colleagues (2022) found that inconsistencies regarding the institution’s COVID policies led to confusion, stress, and safety concerns among the staff who described the conditions as chaotic. As stated by one participant, “we had no idea what was going on, and it just felt like we were playing catch up all the time” (p. 10).

Relatedly, Schultz and Ricciardelli’s (2022) interview data with twenty-one Canadian federal prison correctional officers demonstrated that rapid shifts in COVID policy led to inconsistencies and tensions that undermined trust and institutional legitimacy. The ever-changing conditions of the pandemic likely brought forward unique hardships and stressors in U.S. prison settings. However, missing from current discourse is an examination of how COVID impacted the forms and functions of DOCs around the United States from the perspective of frontline correctional staff (Puglisi et al., 2023).

### Prison operations and (bounded) decision-making

We draw on bounded rationality theory within a correctional environment to provide a foundation for considering the impact of COVID on the forms and functions of DOCs across the country. Bounded rationality (Simon, 1953, 1956), or the idea that people often make decisions based on limited time, information, and cognitive processing abilities, allows us to understand how a “shock to the system,” like COVID, can influence the spur-of-the-moment decisions faced by correctional staff—from line staff to wardens—and the delivery of correctional services. During day-to-day operations most people rely on heuristics which are cognitive processes that allow for efficient decision-making, minimizing the consumption of a person’s limited processing capabilities (Gigerenzer & Gaissmaier, 2011). While correctional staff operate under the constraints of institutional rules and regulations, they often must make quick decisions based on the information presented to them; for example, how to handle rule infractions considered criminal (e.g., assault) to minor acts that interfere with the daily prison routine (e.g., indecent language). Correctional staff also face situations where misconduct (e.g., harassment, sexual violence) is perpetrated by fellow officers (Novisky, Narvey & Piquero, 2021). It is important to highlight that decision-making—something especially salient to the use of heuristics—is not performed in a vacuum. Rather, the context in which a person finds themselves plays a pivotal role in the decision-making process (Gigerenzer, 2004; Simon, 1956).

A key mechanism of bounded rationality is the role of emotions in decision making. Some argue that emotions are antithetical to rationality (Holmes, 1995), while other scholars have argued that emotions are a neglected but crucial part (e.g., Hanoch, 2002; Kaufman, 1999). Emotional arousal can determine the saliency and importance given to certain tasks. When disruptions occur, emotions are an essential pathway for the development of coping strategies and problem solving mechanisms, particularly when faced with limited time and information, as “they signal [to] the human agent that an important goal needs attention” (Kaufman, 1999, p. 136). It is not uncommon for line level workers to cope with demands that exceed the time and resources they actually have available to them (e.g., Maynard-Moody & Musheno, 2000; Tummers et al., 2015). These demands, in turn, promote the use of simplified decision-making through heuristics. However, this can result in policies “in action” being divergent from policies “on paper” (Gofen, 2014).

The onset of COVID presented an opportunity to explore the impact such a disruption could have on decision-making, as prisons had to quickly adjust and adapt how they delivered services and interacted with incarcerated people. Health and safety regulations were constantly being updated, causing confusion and disruption to day-to-day operations. Indeed, Ferdik et al. (2022) reported that “officers felt confused as to what was being expected of them, or what would possibly change again the next day” (p 10). Previous experiences, perceptions towards information, and how information is presented, among other things, can impact how workers allocate their resources and process information (Moseley & Thomann, 2021). As such, the pandemic created a context conducive to both heightened emotions and high stakes decisions (Pedrosa et al., 2020). For example, being present when someone sneezes may trigger fear, apprehension and cause an individual to prefer keeping a safe distance, even during work-related tasks that require proximity (e.g., conducting counts, searches, or transfers).

With this in mind, we explore decision-making during an emotionally charged time (the COVID pandemic) to unpack perceptions of operational response efforts among correctional staff. To do so, we rely on a mixed methodological research design, a survey and focus groups, both of which were developed in tandem among a broader collaborative. Mixed-methods research “helps to understand the holistic picture from meanings obtained from interviews or observation to the prevalence of traits in a population obtained from surveys, which add depth and breadth to the study” (Wasti et al., 2022, p. 1176). This approach was well-suited to our goals as we aimed to (1) provide information on the issues faced by correctional staff while responding to COVID and (2) understand how correctional staff were interpreting their understanding of how COVID impacted prison operations. To be clear, we are not explicitly testing bounded rationality but using it as an organizing framework for our findings.

## Methods

### Surveys of correctional agency officials

The purpose of the quantitative portion of the study was to determine the issues correctional agencies faced in responding to COVID. To do this, we surveyed state correctional agencies in collaboration with the National Institute of Corrections (NIC) and the Correctional Leaders Association (CLA). The survey was administered electronically via Checkbox software between April and May 2021. We focused on this period because enough time had passed from the onset of the pandemic to understand correctional responses, impacts, and lessons learned, but not too much time to undermine recall. Additionally, this timeframe allowed us to obtain data on vaccinations, which were first administered to incarcerated individuals in January 2021 and become more accessible in prisons in April 2021. Initial survey invites were e-mailed via CLA to contacts in all 50 state agencies, as well as a follow-up email two weeks later encouraging participation. We also made targeted contacts with nonrespondents until the survey was closed in May 2021. Our efforts resulted in a response rate of 62 percent (31/50 state agencies). Ongoing litigation, perceived survey duration, and limited resources prohibited some agencies from completing the survey. There was sufficient variation by region of the country and size of the custodial population (see Appendix A for a list of participating agencies). About three-fifths of the total U.S. state custodial population were housed in the agencies that participated in the survey. All study procedures were approved by the Institutional Review Board at CNA.

The development of the survey was informed by advisement offered by the CDC’s “Interim Guidance on Management of Coronavirus Disease 2019 (COVID-19) in Correctional and Detention Facilities.” While the CDC has issued over one dozen updates to their guidance over the course of the pandemic, this survey was based primarily on the guidance issued on October 21, 2020. As noted above, the guidance was organized around the themes of operational preparedness, prevention of transmission, and clinical management. Our instrument, which contained 104 items within 13 domains, was composed primarily of discrete questions. Within each agency, many individuals contributed to completing the survey. The typical agency had input from four different offices or groups, the most common of which were the research office (*n* = 16), central office (e.g., director, deputy director; *n* = 40), and medical staff (*n* = 12).

We report information on counts of the custodial population and security and non-security personnel at the beginning of 2020 and 2021, as well as cumulative counts of infections and deaths as of survey completion date (i.e., April/May 2021). Other examples of items addressed in the survey included operational preparedness and impacts; staffing challenges; preventative and management measures undertaken; communication practices with public health, employees, and incarcerated people; and reliance on existing or new policies concerning communicable disease. Simple descriptive statistics are reported for each of the areas for cases with valid responses.

### Focus groups with correctional personnel

Qualitative data were gathered using focus group methodology. An interview guide was developed to structure the topics covered across the focus groups, but a conversational tone was implemented to prioritize in-depth discussion and rapport building (Hesse-Biber & Leavy, 2011). The interview guide (available upon request), which was the same across all focus groups, consisted of 11 open-ended questions that targeted how agencies managed and adapted to achieve continuity of operations during the first year of the COVID pandemic. The focus group moderator (the first author) incorporated follow-up prompts and clarifying (non-scripted) questions to help facilitate a natural flow of discussion.

The focus group sample was drawn from a curated list of staff who occupied at least one of five DOC roles (i.e., Directors and Deputy Directors; Human Resources and Training; Custody and Support Staff; Medical and Behavioral Health; Wardens and Deputy Wardens). The sampling frame, an e-mail list of those who met the criteria, was generated by CLA, and then provided to the NIC for use in this research. Respondents on the list were contacted via targeted e-mail invitations. These e-mail invitations were stratified by staff type, with the goal of organizing separate focus groups for each of the five staff DOC roles outlined above. An initial e-mail was delivered to individuals inviting them to participate in the appropriate focus group. These efforts were followed up with additional e-mails over several weeks to encourage participation. Our goal was to secure approximately 7–10 participants per focus group across the five focus groups to ensure data saturation would be met (Glaser & Strauss, 1967; Guest et al., 2017). When the number of willing participants exceeded the desired focus group size, efforts were made to prioritize selection of participants to diversify the sample by geographical region and gender. When the number of willing participants fell short of meeting our desired focus group size, more targeted contacts were made with non-respondents to help increase participation. No incentives were provided for participation.

All focus groups were completed via Zoom between July and September of 2021. This timeframe notably coincided with the rise of the Delta variant. Each focus group lasted an average of 70.2 min, was audio-recorded, and transcribed verbatim. These methods yielded approximately 216 single-spaced pages of data.

The final sample includes 62 respondents across five focus groups, including 11 individuals in the Directors and Deputy Directors group, eight in the Human Resources and Training Group, seven in the Custody and Support Staff group, 16 in the Medical and Behavioral Health group, and 20 in the Wardens and Deputy Wardens group (see Table 1 for descriptive data). A total of 22 state DOCs were represented across the focus groups. Participation was secured from all regions of the county, along with larger (e.g., CA, TX) and smaller (e.g., TN, VT) prison systems. The majority of focus group respondents were male (*n* = 37; 59.67 percent) and had worked with the DOC for an average of 21.75 years.

**Table 1:** Descriptive data for focus groups

	Mean or *n*
Mean length of focus groups (minutes)	70.2
Participant roles (*n*)	5
Director or deputy director	11
Human resources and training	8
Custody or support	7
Medical or behavioral health	16
Warden or deputy warden	20
Participants’ mean tenure with DOC	21.75
Participant gender (*n*)	
Female	25
Male	37
*N* of U.S. states represented	22
*n* of participants by state	62
Arkansas	1
California	2
Colorado	3
Connecticut	9
Iowa	1
Illinois	4
Kentucky	4
Louisiana	3
Massachusetts	5
Maryland	1
Minnesota	2
North Carolina	5
New Mexico	1
New York	3
Oklahoma	1
Pennsylvania	4
South Carolina	1
Tennessee	1
Texas	3
Vermont	1
Washington	6
Wyoming	1

Following transcription, all data were de-identified and analyzed with NVivo v.12 (NVivo, 2018). Data analysis was grounded in participants’ views (Charmaz, 2014). Open coding was used first to initiate the inquiry and develop a broad list of exhaustive themes that emerged in the data (Glaser & Strauss, 1967; Strauss, 1987). Data were then reviewed again following a process of refined or secondary coding to expand the initial list of themes into more meaningful categories and to eliminate any overlapping codes. Following the process of secondary coding, all codes identified as most prominent were selectively coded (Loftland et al., 2006). A Microsoft Excel spreadsheet was also used to track and record patterns in the data throughout the analysis.

## Results

The custodial population of 31 prison systems participating in the survey was reduced from 705,000 at the beginning of 2020 to 582,500 at the beginning of 2021. This constitutes a reduction of over 17 percent. Every prison system reported a reduction in their custodial population, ranging from 7 percent (Nebraska) to 33 percent (Tennessee). There was also a drop in the number of employees, though not nearly as drastic as the custodial population and, in fact, a few states reported increases. Security personnel fell by 3.4 percent, a reduction of about 4,500, while non-security personnel fell by 2.1 percent, a reduction of about 2,000 (custodial population and employee counts are summarized in Appendix A; custodial population reductions are illustrated in Appendix B). The disease took hold quickly, with all but two agencies (i.e., New Mexico and Wyoming) reporting the occurrence of their first confirmed infections by April 2020. At the time of the survey, around one year into the pandemic, the participating agencies indicated that about 203,000 incarcerated people and 63,400 employees had been infected, while nearly 1,250 incarcerated people and 120 employees had died from the disease. These numbers are slightly lower than expected based on other sources (e.g., Carson & Nadel, 2022). That said, there are very few examples of state correctional agencies experiencing such universal changes in and impacts on their custodial and employee populations in such a short period of time, which is a useful starting point to understand the views of correctional personnel.

Respondents collectively agreed that COVID had drastically impacted daily operations at their respective institutions. When focus group participants were asked to summarize these impacts in just a few words, they chose words like “profound,” “daunting,” “complicated,” “extremely challenging,” “distracting,” and “circus-like.” A contributing factor involved the unprecedented nature of the circumstances imposed by the pandemic, as well as pandemic-response guidelines that shifted frequently. One respondent compared their working conditions to “trying to fly a plane while also building it.” Another respondent described conditions as “hypersensitive.” As stated by the participant, “if someone *even cleared their throat*, we were sending them to get tested just to make sure [it wasn’t COVID].” These excerpts from the focus groups highlight the complicated, strenuous, and emotional contextual environment in which correctional employees were forced to make decisions.

Participants’ descriptions of their working conditions reflected feelings of stress, exhaustion, and frustration. Despite the acknowledged hardships of working in corrections during the pandemic, however, respondents also relayed feelings of pride given their abilities to quickly problem solve, implement, and sustain pandemic response efforts. Throughout the data, three areas emerged as central to pandemic operations: challenges in staffing; implementation of public health measures; and changes to programs and services. We address each of these areas next, followed by a summary on “lessons learned” by correctional agencies during COVID.

### Challenges in staffing

The most apparent operational hardship that emerged among correctional staff was staffing shortages. Table 2 provides a sense from the survey data of how agencies perceived the issues. Hiring new employees was identified as a major problem by half of the agencies and as a moderate problem by 21 percent of agencies. Employees calling in sick or caring for sick family members was also burdensome (calling in sick: moderate or major problem = 79 percent of agencies; caring for family members: moderate or major problem = 71 percent). The homeschooling of children was less of a challenge, though half of the agencies stated this was still a moderate or major problem. There seemed to be a split in employee retention, as fewer than half of the agencies perceived it as a moderate or major challenge. Still, it was the second-most endorsed of the major problems in staffing after hiring.

**Table 2:** Challenges in staffing

	Valid *N*	*How challenging have the following issues been for your agency?*							
		Not a Problem		Minor Problem		Moderate Problem		Major Problem	
**Issue**		%	(*n*)	%	(*n*)	%	(*n*)	%	(*n*)
Employees call in sick or taking leave	27	4%	(1)	19%	(5)	53%	(14)	26%	(7)
Employees caring for sick family	28	4%	(1)	25%	(7)	50%	(14)	21%	(6)
Employees homeschooling children	28	11%	(3)	39%	(11)	32%	(9)	18%	(5)
Hiring new employees	28	11%	(3)	18%	(5)	21%	(6)	50%	(14)
Retaining employees	27	22%	(6)	30%	(8)	11%	(3)	37%	(10)

Many focus group participants explained that they were operating at significantly reduced staffing capacities, which added to the complexities of COVID response efforts; a hardship identified in other studies in the United States (e.g., Ferdik et al., 2022). Some acknowledged staffing shortages were already problematic pre-COVID, and only got worse during COVID. Some drivers of staffing shortages included hiring freezes and interruptions in staff training academies and hiring initiatives. As one respondent explained:

“We had to cease our academy training, so we missed out on multiple classes. All in all, once we did we resume, we were [at] half [staffing] capacity. We were about 1,300 staff down as far as what we would have hired had it not been for COVID…we were already short staffed going into COVID and now, not being able to have academy classes for four months…and then once we did start, operating at half capacity – it’s going to take us several years to come out of that.”

Another focus group participant summarized:

“We were in a really bad spot recruitment and retention wise, and we were wrapping up a recruitment and retention project when COVID hit us. All of the things that the committee recommended had to be put on hold. Things that we were doing to try to improve our ability to hire people didn’t get to be implemented as we’d like to have.”

Staffing shortages along with turnover are added burdens to correctional officers’ already strained resources during their day-to-day decision-making process. Other drivers of staffing shortages cited by respondents included staff call-offs, quarantines, spikes in retirements, burnout, declines in mental health and morale among staff, and an inability for correctional agencies to offer competitive salaries given pandemic conditions, particularly among nursing staff. As one participant explained:

“Our governor decided that our correctional officers weren’t first responders. And so our correctional officers were set to get vaccinated according to their age bracket. When you have these folks that are literally not going home because they don’t want to get their families sick, and on top of that, you then tell them you don’t value them, you can imagine what that does to morale.”

Respondents in the focus groups perceived the general lack of community support correctional staff received during COVID as another source of staffing shortages. This lack of support appeared to foster frustration and other negative emotions that contributed to the staff shortages experienced by correctional agencies:

“One of the biggest issues was the lack of empathy for staff that we have, that work in our agency every day…whereas everybody else in the community was shown as being ‘heroes,’ with our staff they were talking about them being the source [of COVID]. Our employees were first line workers, they went into COVID positive environments daily and weren’t recognized for that. That was a real struggle.”

Participants emphasized how trying these circumstances were for staff. Knowing the impact of infection and the concern for their families resulted in staff making decisions they thought of as adequate, but not optimal. As explained by one respondent:

“It’s traumatic what they [staff] saw and what they went through. The choice between going to work and to your family, and making that entrance back into a household. We had, just like all of you [points to other participants]…staff sleeping together in their campers in the parking lot because they didn’t want to go home to their infant children.”

Staff explained that the trauma of working through the pandemic has been multi-faceted in that staff have been expected to perform risky work, witness the deaths of colleagues and residents, take on new job responsibilities, and receive very little recognition in the process, all while under constrained decision-making circumstances and heightened emotional states (e.g., decisions such as sleeping in campers out of fear). These findings are especially concerning in light of research that indicates that stress on the job is related to poorer mental health and operational outcomes for correctional officers (Frost & Monteiro, 2020; Worley et al., 2022). Furthermore, adding job duties to correctional officers requires them to further consider the best way to expend their limited resources (e.g., time devoted to these duties). Related to medical staff, one focus group participant explained:

“We’re seeing the fallout…we’ve lost a significant number of our nurses in the last couple of months. And every single one of them has listed ‘the stress of COVID’ as being part of the reason that they’re looking for alternative work.”

This summary highlights that the added stress of a significant disruption can shift what people perceive as a satisfactory decision. Unemployment is rarely, if ever, optimal. However, in the context of COVID, it was a satisfactory decision for many of the staff. These staffing challenges were compounded by the strain of increased public attention towards how institutions were handling the pandemic. Summarized by one staff member:

“I think for the agency a big challenge was the external interest in what was happening. So many community organizations and outside people really wanting to know. For me, it was about data…but it was really about people wanting to understand what was happening and they wanted it quick and fast. And we had no standards for what we were capturing…our staff did a really tremendous job in building a database so we could start capturing who was tested, when they were tested, was it a retest, was it a first positive, a second positive, when were they vaccinated, what kind of vaccination did they have? Those kinds of systems did not exist, yet that was the information people wanted…And so there was that added external pressure that we don’t see on a normal basis to really put a microscope on our operations at the same time we were trying to figure it out. For us, that was a huge challenge.”

Respondents felt that despite their best efforts given rapidly changing conditions and recommendations, the public–and in particular advocacy groups and the media–were unfair in their summaries of the decisions made and circumstances within institutions, which led to feelings of demoralization and frustration. As explained by one focus group respondent:

“It’s a time in corrections probably where we were more transparent than ever before, and we got beat over the head with it in every state. As we provided information on what was going on in our institutions, the media just took it and ran with it and it made it easy for them to write negative stories, much more negative than usual. You couldn’t get a break.”

Participants emphasized that these depictions of correctional workers were largely negative, despite massive efforts by correctional agencies to implement public health measures and respond to COVID relief efforts, including overseeing the mass production of PPE (e.g., masks, sanitizer, gowns) for communities across the country. Again, participants felt that given the context of the situation, (bounded) decisions made by correctional staff resulted in satisfactory outcomes that were left unacknowledged.

### Implementation of public health measures

Table 3 provides a summary of the public health measures implemented by correctional agencies in their facilities to help prevent COVID spread, as reported in the survey. Screening of incarcerated people and employees was prevalent (93–100 percent of agencies). Testing was also quite common, though more common among incarcerated people than employees upon contact with suspected or confirmed cases and when showing symptoms (incarcerated people = 71 to 96 percent; employees = 41 to 59 percent). Quarantining was used primarily at intake (96 percent) and among those infected (100 percent); fewer agencies quarantined people when they were to be released or transferred (57 percent). Agencies distinguished their masking policies based on incarcerated people and employees, always requiring masks for employees but only in common spaces for incarcerated people. Vaccination availability was highly prevalent for incarcerated people (100 percent) and employees (96 percent).

**Table 3:** Preventive measures: screening, testing, quarantining and masks

		Yes		No	
	Valid *N*	*%*	(*n*)	%	(*n*)
**Screening**					
Incarcerated people					
At intake, self-reported symptoms	29	93%	(27)	7%	(2)
At intake, temperature checks	28	96%	(27)	4%	(1)
At release, self-reported symptoms	28	96%	(27)	4%	(1)
At release, temperature checks	28	96%	(27)	4%	(1)
Employees					
At entry, self-reported symptoms	29	100%	(29)	0%	(0)
At entry, temperature checks	28	96%	(27)	4%	(1)
**Testing**					
Incarcerated people					
Conduct testing	29	100%	(29)	0%	(0)
Upon entry to facility	28	82%	(23)	18%	(5)
Upon contact with suspected cases	28	71%	(20)	29%	(8)
Upon contact with confirmed cases	28	96%	(27)	4%	(1)
Showing symptoms	28	96%	(27)	4%	(1)
Voluntary request	28	96%	(27)	4%	(1)
Upon release from prison	28	79%	(22)	21%	(6)
Employees					
Conduct testing	29	93%	(27)	9%	(2)
Upon contact with suspected cases	27	41%	(11)	59%	(16)
Upon contact with confirmed cases	27	59%	(16)	41%	(11)
Showing symptoms	27	52%	(14)	48%	(13)
Voluntary request	28	96%	(27)	4%	(1)
**Quarantining**					
Use quarantining for incarcerated people	28	100%	(28)	0%	(0)
Admission to facility	28	96%	(27)	4%	(1)
Release or transfer	28	57%	(16)	43%	(12)
Suspected/confirmed cases	28	100%	(28)	0%	(0)
Contact with suspected/confirmed cases	28	93%	(26)	7%	(2)
Use contact tracing	28	93%	(26)	7%	(2)
**Masks**					
Incarcerated people					
Masks encouraged, but not required	28	0%	(0)	100%	(28)
Masks required only in common areas	28	39%	(11)	61%	(17)
Masks required at all times	28	61%	(17)	39%	(11)
Employees					
Masks encouraged, but not required	28	0%	(0)	100%	(28)
Masks required only in common areas	28	7%	(2)	93%	(26)
Masks required at all times	28	93%	(26)	39%	(2)
**Vaccinations**					
Offered to Incarcerated people	28	100%	(28)	0%	(0)
Offered to Employees	28	96%	(27)	4%	(1)

As reported in the focus groups, correctional agencies faced multiple barriers that made it difficult to implement these public health guidelines, which had the perceived effect of minimizing progress towards pandemic responses despite substantial workload efforts to the contrary. One example raised was the difficulty of effectively isolating people given population sizes, halts on transfers, and information delays. As one respondent explained:

“Our dormitories were going at an amazing rate of COVID positives once they started, so we were trying to isolate those folks and get people transferred out of our facilities to other facilities, but transfers were put on hold because of the pandemic…so we had to try to manage it our own. Things like that were difficult, especially in the beginning when there was not a lot of information or not a lot of testing available.”

Our survey data affirmed this. Transfers reduced within and between states in 93 and 89 percent of the agencies, respectively. In fact, one-quarter of the agencies indicated that cross-state transfers ceased altogether, not just reduced, while 14 percent stated that intra-state transfers ceased altogether.

Focus group respondents also emphasized architectural limitations as barriers to implementing quarantining and social distancing. One participant surmised:

“We were very limited on space, and we had a lot of older dorm style settings. We just didn’t have the room to be able to social distance and isolate.”

What makes observations like this even more striking is that they were made in the presence of large-scale year-over-year reductions in the custodial populations (Carson, 2021a, 2021b). This excerpt also highlights the active disjoint between policies in “practice” and policies on “paper.” While there was an on-the-paper requirement to social distance, it was not a realistic option to implement in practice. Therefore, issues like these strain correctional officers’ ability to meet health regulations while carrying out their duties, pushing them to find solutions that are merely “good enough.”

Staff also referenced other factors that complicated the implementation of public health measures, including poor masking compliance and vaccination resistance, at times from both incarcerated people and staff. Focus group participants believed masking and vaccine avoidance among the incarcerated population was at least partially driven by distrust between incarcerated people and staff, which has been referenced as a reason incarcerated people have refused or been hesitant to receive the vaccine in other research (Schultz & Ricciardeli, 2022; Stern et al., 2021). Respondents acknowledged that baseline-levels of distrust among incarcerated people towards the criminal justice system were likely exacerbated due to the constantly changing circumstances of the pandemic, as well as the limited abilities incarcerated people had to access real time information from outside prison walls. In line with the arguments of bounded rationality, incarcerated individuals were making the most optimal decisions possible, while faced with feelings of distrust and a lack of or consistently changing information.

Participants agreed that policy development in this area required creativity due to hesitancy among incarcerated people to receive vaccines and follow other COVID protocols. Participants acknowledged that further reducing the already limited freedoms and privileges of incarcerated people was a complex challenge, as choices in prison are already so limited (Crewe et al., 2022; Sykes, 1958). One strategy raised as an example to address this dynamic was the incorporation of peer ambassadors and monitors to help with policy-related buy-in and information sharing. As described by one focus group respondent:

“That worked really well here. Even amongst the mentally ill population, we had ambassadors who were designated as seriously mentally ill and went in and had conversations with others. I think that's one of the reasons our compliance rate for our individuals in custody is so high. We also got our monitors from all our various lawsuits to do video PSAs. Because a lot of times we find that, you know, individuals in custody kind of identified more and had more trust with the monitors because they're here to watch us, right? We ran those PSAs constantly on loop, in all of our institutions and that also helped.”

Therefore, the overall goal of this initiative was met: sharing information with incarcerated individuals from a trusted source enhanced decision-making abilities. Staff shared that implementing new–and often changing–COVID policies also required fundamental cultural shifts within corrections, especially as they related to PPE. For example, one respondent explained:

“We pushed the masks real hard for the staff and inmate population. It was difficult at first because in the prison system, we don't like the inmates to have masks. It was a culture change for all of us. And now everybody's got to wear a mask and that didn't come out right away…everything came out piecemeal.”

Indeed, it is one thing to require masks, but much different to enforce their use. Forty percent of agencies surveyed said incarcerated people disobeying COVID protocols was a moderate or major problem, compared to 21 percent for employees. To help address compliance rates among incarcerated and employee populations, focus group respondents explained steps were made to address information sharing. For example:

“One of the biggest challenges was the lack of communication—due to roll call being stopped—to our frontline staff and the ability for them to ask questions. This was also an issue with the inmate population. To rectify this, we installed monitors in the officers’ breakroom just for staff awareness and notifications related to the pandemic. We also conducted numerous tours and held community forums in an attempt to relay the necessary information and to be there for staff.”

Participants agreed that because of the circumstances of the pandemic, they had to be open-minded, flexible, and quick to implement new information as it came in. This also meant that policy responses shifted frequently, sometimes as drastically as from one shift to the next. Our findings reinforce those by Schultz and Ricciardeli (2022), who found that correctional officers in Canadian prisons were similarly overwhelmed by rapidly changing policy directives. As stated by one of the study’s participants, “It’s like, ‘Okay so what do we follow now? No idea.’ We would ask inmates, ‘What’s the new rules? You guys seem to know more than us, so what’s the new rules now?’” (p. 7).

Our focus group respondents described efforts to update COVID and emergency response handbooks, only to have to go back and modify them again soon after. Forty-six percent of agencies surveyed said they were updating employees with communications multiple times per week, while another 33 percent stated this was occurring multiple times per month. And every agency provided training on the disease, its symptoms and transmission, along with use of PPE and COVID-related protocols. Most respondents agreed that these circumstances translated to noticeable increases in workloads for the duration of the pandemic, frustration among both staff and incarcerated people, and reductions in sleep among staff.

These examples demonstrate that, perhaps unlike in other criminal justice areas (Piquero, 2021), there was an abundance of information sharing within corrections. However, too much information sharing could foster an over-emotional arousal—for example, high levels of frustration or eventually apathy—impacting the ability to make decisions. Additionally, sharing information with incarcerated people was seen as critical by staff as a way to shift the decision-making process to those reluctant to wear masks or get vaccinated.

### Changes to programming and services

Collectively, participants agreed that COVID dominated daily operations at facilities. As summarized by one focus group respondent:

“I can say that I’ve focused less on my normal job and more on COVID and COVID policies and let everything else kind of go. I wasn’t allocated more time to do anything…we were all just focused on either COVID or COVID related things, and just kind of the stuff we would normally work on would go by the wayside a little bit.”

This example illustrates the idea that during times of uncertainty (e.g., COVID) correctional staff may be asked to carry out daily tasks through times of resource deficit. This leads to the development of heuristics, as decisions need to be made quickly to offset the lack of time allocation. However, because of the ever-changing circumstances, newly developed heuristics became obsolete, further straining individuals’ processing power and decision-making.

Disruptions in programming and services were especially apparent. Agencies recognized that cuts were made, which are summarized by the survey data reported in Table 4. Work details, especially those occurring offsite of facilities, were heavily curtailed. Eighty-six percent of agencies stated that programming was reduced by a little (50 percent) or a lot (36 percent). Interactions with other incarcerated people were also restricted by reducing time in the day room (53 percent) or recreation (67 percent). There were some increases to compensate, such as more medical services (48 percent), reading materials (47 percent), and tablet (33 percent) and television (32 percent) time made available, but few would argue this was comparable in meaning and impact to social interaction and programmatic services.

**Table 4:** Changes to activities, programs and privileges

				Complete Suspension		Partial Suspension		No Suspension			
**Work Detail**	Valid *N*			%	(*n*)	%	(*n*)	%	(*n*)		
Inside of facility	28			4%	(1)	93%	(26)	4%	(1)		
Outside on facility grounds	28			25%	(7)	75%	(21)	0%	(0)		
Offsite of the facility	28			64%	(18)	32%	(9)	4%	(1)		
		A lot less		A little less		Equivalent		A little more		A lot more	
**Services/Privileges**	Valid *N*	%	(*n*)	%	(*n*)	%	(*n*)	%	(*n*)	%	(*n*)
Medical services	27	0%	(0)	11%	(3)	41%	(11)	22%	(6)	26%	(7)
TV time	28	4%	(1)	4%	(1)	61%	(17)	7%	(2)	25%	(7)
Reading materials	28	0%	(0)	0%	(0)	54%	(15)	36%	(10)	11%	(3)
Commissary	28	0%	(0)	14%	(4)	61%	(17)	14%	(4)	11%	(3)
Tablet time	21	0%	(0)	5%	(1)	62%	(13)	14%	(3)	19%	(4)
Day room	28	14%	(4)	39%	(11)	36%	(10)	7%	(2)	4%	(1)
Recreation	28	21%	(6)	46%	(13)	25%	(7)	7%	(2)	0%	(0)
Programming	28	36%	(10)	50%	(14)	11%	(3)	4%	(1)	0%	(0)

During the focus groups there were many references made to educational and vocational programming, mental health and substance use disorder treatment, family visitation, and attorney visits. One participant surmised:

“We have found it to be challenging throughout the pandemic to continue to deliver the primary essential mental health services that are required. It’s been difficult because staff call offs have occurred and institutional lockdowns have occurred…because of the virus spread our clinical staff are pulled from their clinical responsibilities and they are now doing the duties that individuals who are incarcerated typically do, like making lunches and feeding the population. We’re unable to do clinical responsibilities that we would typically do and it has become a ‘dance of triage’ that we’ve become very accustomed to.”

Participants emphasized it was not one, but multiple operational aspects of programming and services that slowed down during COVID. These findings are consistent with international research that reported significant disruptions in programming and services that altered the landscape of daily prison life (Maycock, 2022). To help mitigate lapses in programming and services, staff were tasked with complex problem solving and creative deployments of resources. For example:

“We had to become creative in how we ensured that our population received the services needed, while also juggling the staff that was either positive and isolated at home, quarantined at home in a system that already has quite a bit of shortages when it comes to correctional staff. So becoming innovative in doing that. Of course, at that point in the beginning our Governor had ceased visitation for family and loved ones with inmates. And at the time our state did not have tablets in place, and we just now are going live [with tablets] in the next few months. We quickly had to pivot and find different avenues to mitigate the loss of that human touch or human contact with visitation. Whether it be through phone calls or…we actually came up with some tablet visitation with some handhelds.”

Beyond maintaining existing programming and services, respondents addressed how COVID influenced the development and implementation of new initiatives. As summarized by one focus group respondent:

“It’s [COVID] been a distraction from the innovations that we were trying to put in place and the things that we were moving forward. When you have to stop everything to focus on COVID and not move people and not bring intakes in, it really shut down the innovation we were trying to deploy.”

Another participant stated:

“Many different things were interrupted. We had rolled out a new risk and needs assessment and that training and its rollout were stopped…we were in the process of also developing our regional re-entry centers, as well as some mission-focused facilities that were halted for the time being…there were several new programs that were designed that we were going to be training the staff to conduct with the individuals in custody; those all had to be stopped. We also had several new University partners that were going to be providing post-secondary education, that had to be stopped. The list goes on and on with what was disrupted with COVID.”

These examples provide valuable context about how shifts brought on by the pandemic are important to understanding decision making by correctional staff at the time. While staff buy-in for new initiatives in our sample was high, for example, they realized that programs they wanted to support were stalled–essentially held in abeyance–based on the massive undertaking of managing the pandemic. While exhausted, respondents were also generally proud of their efforts to identify and implement creative and targeted solutions to complex problems during the first year of the pandemic. In a time when participants felt they were being overburdened with new tasks, constant changes in available information, and a resource deficit, they felt they handled the situation as best they could. These efforts resulted in careful thought and attention among participants towards broader lessons learned for prison management. In the final section, we review the data on policy considerations moving forward.

### Lessons learned

Table 5 outlines how agencies crafted their policy responses to COVID based on the survey data. Consistent with the CDC’s operational preparedness guidelines, there was a great deal of communication with public health. Nearly two-thirds of agencies said they communicated daily with local or state public health boards, while the remaining agencies said they communicated weekly or monthly. Sixty-four percent of the agencies said they implemented the National Incident Management System (NIMS), which permitted collaborative cross-agency critical response capabilities to respond to the pandemic. Owing to the unique challenges presented by the pandemic, very few agencies indicated their existing communicative disease policies were sufficient. Thirty percent of agencies devised entirely new policies to respond to the pandemic while 63 percent relied on a combination of new and existing policy.

**Table 5:** Policy responses to COVID

		Yes		No	
	Valid *N*	%	(*n*)	%	(*n*)
Reporting to public health board	31	97%	(30)	3%	(1)
Communicate daily	28	64%	(18)	36%	(10)
Communicate weekly/monthly	28	36%	(10)	64%	(18)
Implemented NIMS	28	64%	(18)	36%	(10)
Policy leveraging					
Relied mostly on new policy	30	30%	(9)	70%	(21)
Relied equally on new/existing policy	30	63%	(19)	27%	(11)
Relied mostly on existing policy	30	7%	(2)	93%	(28)

The focus group data reinforce these collaborative efforts. Respondents believed that the success of maintaining operations during the pandemic was intimately linked to collaborations with other personnel, institutions, agencies, and community partners. As described by one respondent:

“It was vastly important…that all of the different disciplines, whether it’s in the county or state system, really had to come together, communicate effectively, and work closely with one another. The pandemic really showed the importance of that.”

Such reflections emphasized the importance of engaging with scientists and public health experts, and further highlights the importance and impact of information sharing on decision-making. One participant described how this looked in practice:

“Our meetings were not just with correctional practitioners. For us, we had the [state] Department of Health epidemiologists on every call…we had that connection to public health, connection to the broader focus of state government. This was really, really important because continuity of message, and making sure that the message got out as quickly as we could get it out, before it turned to rumor.”

Another respondent explained further:

“One of the things we learned is that teamwork was very important. As we worked with our partners from the [state] Department of Health, we brought them in to the fold very early on, and they visited with us on a weekly basis with our epidemiologists. They looked at every quarantine situation, they staffed critical staff with our nurses, every incident that we had of COVID early on and throughout the pandemic…they’ve worked really well with us in terms of sending nurses to assist us, they worked with us on vaccination clinics down the road. We found that working with those partners really made a big difference for us.”

Thus, moving forward, respondents believed that such collaborations are important to continue to nurture and expand upon. From the perspective of participants sampled in our study, agencies should be committed to fostering a culture of interdisciplinary collaboration, networking, and information sharing.

Another lesson learned was that even though pandemic circumstances were perceived as stressful, respondents generally felt the pandemic became a catalyst for necessary and continuing expansion and growth. As explained by one focus group participant:

“We became very cognizant that our policies were outdated, and that we had not really taken a sharp keen eye and taken a look at them. This [COVID] created a whole new policy initiative for us, not only did we have to create new policies related to COVID, we had to look at our existing policy structures, some of which hadn’t been updated since the 90s. Now we have a structure that we are analyzing each one of our policies annually. It really made us take a look at, ‘okay, what really works for corrections now, during COVID, as well as post COVID?’ It was an eye-opening experience when it comes to policies for us.”

**Fig. 1:**
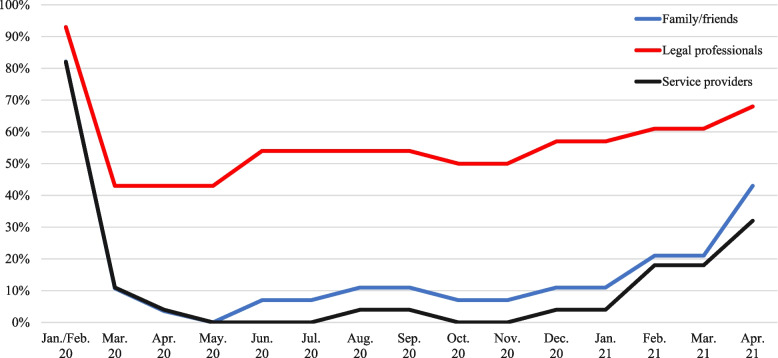
Changes in access to correctional facilities

The pandemic also motivated meaningful and useful expansions in technology. Survey data in Fig. 1 shows that by May 2020, access to facilities was completely shut off to visitors and volunteers. One year into the pandemic, the majority of agencies reopened, which was prior to the outbreak of the Delta and Omicron variants. As one focus group respondent explained, this was largely possible owing to technological advancements:

“It [COVID] forced us to look at ways to do things differently and more efficiently with things like Zoom, video visiting. We had never offered that before, which hasn’t been real popular, but it’s forced us to look at some things. We were piloting to do some self-reporting using technology in our supervision population. So because we had the pilots in place, we were able to turn those on….with technology, I think it forced us to look at new ways to do things and not just stay where we were.”

Focus group respondents offered multiple examples of how they turned to technology to help them become more adaptive to pandemic circumstances. This included a wider implementation of video visits with families and attorneys, expanded use of tablets for recreation and programming, remote court hearings, and virtual correctional officer academy trainings. The survey data confirm there was a major increase in virtual court appearance in 54 percent of the agencies and a moderate increase in 19 percent. There were also increases in the number of phone calls (68 percent), minutes per phone call (48 percent), and number of video visitations (82 percent) made available to incarcerated people; no agencies reported a concomitant increased in costs of phone calls or video visitations. In fact, 67 percent and 45 percent of the agencies stated that these costs dropped, respectively, while the rest stated that the costs remained unchanged. Respondents agreed that efforts towards expansions in correctional technology should continue given their demonstrated value during the pandemic.

Other aspects of correctional operations were also identified as needing attention. More specifically, focus group participants stated that expansions in capacity, staffing, supplies, and resources were critical. One respondent offered the following example related to staffing:

“It's always been hard to hire nurses, there's a national shortage and particularly in the field of corrections. I think to ensure that we all have adequate medical staff and have the ability to recruit and retain, whether it's a push from the federal government or preferably at the federal level, to recognize corrections as an area of critical staffing when it comes to medical titles and nursing, and to run incentives, whether it's tuition forgiveness or loan forgiveness, things such as that. Because we can't move as nimbly as the community and the private sector, which is giving signing bonuses and stealing nurses in that regard. You get involved in public safety because you have a commitment to public safety or public service, you're not in it for the money. But to give us a leg up in attracting those individuals as we go forward in everyday operation, but particularly when we have a health emergency, I think it's an area that gets overlooked.”

Respondents emphasized that staffing initiatives were incredibly important as a priority area, as many agencies were understaffed with high turnover rates. Participants also believed it would be helpful to have initiatives in place to enhance the availability of PPE supplies. All but three agencies said that incarcerated people were manufacturing PPE, primarily masks (96 percent of “yes” responses), but also gowns (64 percent), sanitizer/disinfectant (52 percent), face shields (44 percent), and soap (16 percent).

Prison architecture was also considered in need of improvement to respond to airborne infectious disease. Participants pointed to the structure of physical buildings, such as the installation and/or repair of HVAC systems, and felt it was important to consider how to address housing moving forward given that crammed dormitory style units were difficult to manage during COVID and community release mechanisms were strained. One focus group participant explained:

“Lord forbid this [COVID] happen again down the road, I think one thing that would be valuable would be to have a general protocol or a basic system in place for the housing of positive cases. What we ran into was for two to three months, we basically had all these inmates and nothing to do with them. We couldn't release them back to the community. We did not have the equipment to provide proper medical care. And there was conversation about bringing in National Guard medical tents. There was conversation about putting inmates out in sally ports. There were conversations about putting inmates in hotel rooms. Maybe some of the states had those plans in place, we just didn't…I think emergency housing for 30 days is something that we will try to correct.”

Statements such as these highlight the desire amongst correctional staff to have initiatives and systems in place prior to the onset of a national crises—like the COVID pandemic—which would help in navigating the tumultuous decision-making process and reducing some of the related stress.

## Discussion

The COVID pandemic has been and continues to be an unprecedented challenge for the American criminal justice system (e.g., Gutierrez & Patterson, 2021; Nix et al., 2021; Piquero, 2021). In response, correctional agencies around the country undertook dynamic changes in the forms and functions of their operations, including implementing social distancing and quarantining, reducing prison populations, and administering viral tests to incarcerated people and staff (Carson & Nadel, 2022; Hummer, 2020; Novisky et al., 2020). Yet, *how* COVID shaped (and continues to shape) frontline prison dynamics remains largely a black box. This gap is especially pressing given that while correctional institutions have largely returned to normal operations, the pandemic is not over, and new disease-related outbreaks will occur in the future. These are situations that exacerbate already stressful circumstances and offer little margin for error.

With this context in mind, our findings provide important insight about correctional decision making and the ways the pandemic has fundamentally shifted prison operations. Using a framework of bounded rationality, we find that daily operations were strained, especially in the areas of staffing, implementation of public health policy, and capacities to sustain correctional programming. A need to make satisfactory, even if less than optimal decisions was heightened (Gigerenzer, 2004), which ultimately led some staff to resign from their positions. While prison systems and correctional staff were under-prepared to respond to the pandemic, they addressed complex circumstances with responsive policy decisions and collaborative problem solving bounded by the context of the situation (e.g., prison environment, health requirements, limited information, and high emotions). These findings meaningfully advance the literature, as they offer the *first and only* national, mixed-methods summary of state DOC pandemic response efforts and perceptions about daily operations among a diverse sample of correctional staff. Even entering into the third year of the pandemic, research that elucidates correctional staff pandemic experiences remains limited, and the work that does exist has focused largely on recruiting small samples of correctional officers (Ferdik et al., 2022; Schultz & Ricciardelli, 2022) or on gathering data about COVID incidence (Puglisi et al., 2023). Conversely, our data capture in-depth experiences from prison medical and behavioral health staff, wardens, human resources and training staff, directors, *and* correctional officers, in addition to survey data about overall nationwide DOC response efforts and interpretations about those efforts. 

Our findings motivate several implications for policy and practice. To be sure, policies that invest in meaningful reductions in prison populations have the greatest potential for increasing the efficacy of future pandemic response efforts. As respondents in our sample explained, without the necessary physical infrastructure to accommodate prescribed social distancing and quarantining, such policies will continue to generate confusion and low odds of success. One study found that across 14 Massachusetts state prisons, increases in crowding were associated with significantly higher incidence rates of COVID (Leibowitz et al., 2021). Alternatively, in one jail study, single celling reduced risk of COVID-19 transmission by more than 50 percent (Malloy et al., 2021). These implications are especially important to consider in light of the aging prison population and elevated risk of COVID-19 mortalities among older adults (Kwan et al., 2022), as well as the complicated nature of the use of isolation in quarantine “where decades of overuse of punitive solitary confinement is the norm” (Cloud et al., 2020, p. 2738). We therefore encourage further policy development on prison downsizing and ensuring humane conditions of confinement, particularly during periods of crisis and with attention to vulnerable subgroups.

As understaffing was identified as a problem area for many DOCs in our research, prison downsizing efforts would not only help to increase the practicality of social distancing and quarantining efforts, but also alleviate some of the strains tied to deficiencies in prison staffing, recruitment, and retention. Coupled with downsizing, expansions in correctional staff capacity will be important to address moving forward. As we can anticipate that additional crises will occur, prisons must be equipped with the staffing levels they need to successfully carry out their responsibilities. Results from our focus groups uniquely emphasize that targeted efforts to address nursing shortages are vital. We caution that without meaningful expansions in correctional staff capacity, it is likely that existing staff will be more prone to burnout and stress, further exacerbating existing staffing crises.

Even when downsizing prison populations and/or expanding staff capacity is unavailable or untenable, correctional agencies can improve institutional experiences and operations by better leveraging technology and enhancing lines of communication. When access to facilities was completely shut off to visitors and volunteers in 2020, new and expanded uses of technology emerged and were incorporated successfully. This included increases in virtual attorney and family visits, virtual court appearances, and the implementation of virtual correctional officer training academies. While in person options should be offered whenever safe and possible (especially regarding family visitation), such technology expansions demonstrate that there is room in corrections for creative technology applications (e.g., Murdoch & King, 2020). We suggest that future scholars and practitioners meaningfully explore such possibilities, especially in situations where prison downsizing is unavailable or untenable.

Communication is critical during times of crisis. Communicative partnership efforts, including interdisciplinary collaboration, information sharing, and networking with agencies and organizations outside of state DOCs should be continuously developed and fostered, not *just* during pandemic times, but *at all times*. When COVID hit, these lines of communication were not properly established and may have, in part, contributed to the tremulous nature of day-to-day operations within the prison environment. However, through quick decision-making processes, nearly every agency in our sample reported at least some degree of partnership with outside agencies that they described as vitally important to their operational responses. Additionally, communication *within* DOCs is another avenue that warrants attention. Staff were quick to point out the rapidly changing situation, lack of information, and feelings of frustration. Some studies have shown that there was a lack of or infrequent information provided to incarcerated people (e.g., Ferdik et al., 2022; Pyrooz et al., 2020), which may have contributed to increasing levels of distrust. But as we highlighted, sharing information was crucial in shifting incarcerated individuals’ mistrust of new policies (i.e., wearing masks). Prison administrators should consider devising an effective plan to proactively communicate clear and regular information to incarcerated people and staff to mitigate negative emotions and ensure a safe and orderly prison environment, as these relationships are “the bedrock of effective and humane prison operations” (Schultz & Ricciardelli, 2022, p. 2).

Still, COVID continues to evolve and shift, and we caution that our data only capture experiences through the summer of 2021. Accordingly, our data cannot speak to correctional staff experiences beyond this time point or to the extent to which prison operations may have shifted as new COVID variants emerged. While we have provided the most comprehensive  picture of frontline prison employees to date, it is possible that state DOCs not participating in the study due to litigation or other reasons may not be represented in these findings. We encourage additional data collection efforts that address pandemic-related correctional staff experiences so that variations across time and place are documented and assessed. Additionally, as the purpose of this study was to capture prison staff perspectives of pandemic response efforts, it was beyond our scope to address what it was like to be incarcerated during the pandemic. We acknowledge that the findings presented in this paper expectedly reflect staff perspectives given that lived experiences were not incorporated in the data collection. For example, staff may have perceived COVID response efforts more favorably than incarcerated individuals or may have been more inclined to point out failures of incarcerated individuals to follow COVID protocols relative to staff failures. We encourage further research that uses surveys, focus groups, and interviews with incarcerated people to expand the work we have presented here.

## Conclusions

“Few doubt that major epidemics and pandemics will strike again and few would argue that the world is adequately prepared” (Fan et al., 2018, p. 129). We want to emphasize that future health emergencies are not the only crises that can benefit from establishing (and reviewing) collaborative policies and practices for managing crisis situations; reducing the size of the prison population; expanding staff capabilities and developing strategies to encourage retention; and creating innovative technological advancements to improve operations and facilitate enhanced communication. Indeed, emergencies, including natural disasters (e.g., Hurricane Katrina; Robbins, 2008), riots (Colvin, 1992; Useem & Kimball, 1991), and staffing stoppages/shortages (Martin et al., 2012), happen far too often within the correctional sphere and agencies may not be prepared (Freeman, 1998). Therefore, we hope that the knowledge and operations established during the COVID pandemic provide fruitful avenues of continued improvements in policy and practice to respond to crisis situations efficiently and effectively.

## Data Availability

The datasets used and/or analyzed during the current study are available from the corresponding author on reasonable request.

## Appendices

### Appendix A

List of Agencies and Changes in the Custodial, and Security and Non-Security Personnel Population Counts from January 1, 2020 to January 1, 2021

**Table:**

	Custodial Population				Security Personnel Population				Non-Security Personnel Population			
	Yearly Count		Yearly Change		Yearly Count		Yearly Change		Yearly Count		Yearly Change	
	2020	2021	Count	Percent	2020	2021	Count	Percent	2020	2021	Count	Percent
Alabama	21,900	17,761	-4,139	-18.9%	1,781	1,991	+ 210	11.8%	1,349	1,272	-77	-5.7%
Arkansas	17,398	14,784	-2,614	-15.0%	3,925	3,414	-511	-13.0%	1,534	1,268	-266	-17.3%
California	117,344	92,087	-25,257	-21.5%	24,525	23,793	-732	-3.0%	33,796	33,173	-623	-1.8%
Colorado^b^	17,777	13,528	-4,249	-23.9%								
Connecticut	12,284	9,094	-3,190	-26.0%	4,492	4,446	-46	-1.0%	1,692	1,634	-58	-3.4%
Hawaii^a^	5,208	4,169	-1,039	-20.0%	1,650	1,400	-250	-15.2%	600	500	-100	-16.7%
Illinois	38,082	29,095	-8,987	-23.6%	8,596	8,301	-295	-3.4%	3,998	3,784	-214	-5.4%
Kansas^a^	9,928	8,723	-1,205	-12.1%	2,066	2,066	0	0.0%	1,261	1,261	0	0.0%
Kentucky	12,221	9,804	-2,417	-19.8%	1,749	1,628	-121	-6.9%	929	1,542	+ 613	66.0%
Louisiana	15,087	13,866	-1,221	-8.1%	3,298	3,063	-235	-7.1%	1,389	1,378	-11	-0.8%
Maine^a^	2,175	1,712	-463	-21.3%	800	800	0	0.0%	400	400	0	0.0%
Massachusetts	7,936	6,569	-1,367	-17.2%	3,579	3,471	-108	-3.0%	2,516	2,575	+ 59	2.3%
Minnesota	9,381	7,593	-1,788	-19.1%	1,603	1,518	-85	-5.3%	2,320	2,181	-139	-6.0%
Nebraska	5,680	5,310	-370	-6.5%	1,161	1,178	+ 17	1.5%	955	960	+ 5	0.5%
Nevada	12,445	11,116	-1,329	-10.7%	1,742	1,721	-21	-1.2%	894	872	-22	-2.5%
New Hampshire	2,464	2,136	-328	-13.3%	380	374	-6	-1.6%	360	372	+ 12	3.3%
New Mexico	6,879	6,034	-845	-12.3%	901	957	+ 56	6.2%	991	973	-18	-1.8%
New York	44,276	34,405	-9,871	-22.3%	18,609	18,171	-438	-2.4%	8,471	8,281	-190	-2.2%
North Carolina^b^	34,469	29,716	-4,753	-13.8%								
Oklahoma	25,077	21,683	-3,394	-13.5%	1,754	1,593	-161	-9.2%	2,627	2,524	-103	-3.9%
Pennsylvania	45,254	38,807	-6,447	-14.2%	9,399	9,398	-1	0.0%	7,089	6,854	-235	-3.3%
Rhode Island	2,573	2,069	-504	-19.6%	943	961	+ 18	1.9%	414	392	-22	-5.3%
South Carolina	18,122	15,726	-2,396	-13.2%	2,761	2,462	-299	-10.8%	2,180	2,215	+ 35	1.6%
South Dakota	3,730	3,159	-571	-15.3%	464	484	+ 20	4.3%	315	318	+ 3	1.0%
Tennessee	1,849	1,245	-604	-32.7%	394	364	-30	-7.6%	149	144	-5	-3.4%
Texas	141,549	120,873	-20,676	-14.6%	24,477	23,273	-1,204	-4.9%	10,589	9,921	-668	-6.3%
Vermont^b^	1,665	1,279	-386	-23.2%								
Virginia	29,258	23,978	-5,280	-18.0%	5,459	5,039	-420	-7.7%	5,920	5,942	+ 22	0.4%
Washington	17,000	14,231	-2,769	-16.3%	3,689	3,562	-127	-3.4%	1,296	1,344	+ 48	3.7%
Wisconsin	23,778	20,121	-3,657	-15.4%	3,947	3,997	+ 50	1.3%	4,964	4,906	-58	-1.2%
Wyoming	2,259	1,812	-447	-19.8%	490	600	+ 110	22.4%	477	388	-89	-18.7%
Mean	22,743	18,790	-3,954	-17.5%	4,808	4,643	-165	-2.0%	3,552	3,477	-75	-1.0%
SD	31,233	25,796	5,659	-6.5%	6,705	6,468	291	7.4%	6,494	6,351	222	14.3%
Overall	705,048	582,485	-122,563	-17.4%	134,634	130,025	-4,609	-3.4%	99,475	97,374	-2,101	-2.1%

Sorted by state in alphabetical order

^a^refers to estimates of personnel counts

^b^no personnel data made available

### Appendix B

Changes in Custodial Populations from January 1, 2020 to January 1, 2021.

## Footnotes

1 These guidelines have been updated throughout the pandemic. For the current guidelines see: https://www.cdc.gov/coronavirus/2019-ncov/community/correction-detention/guidance-correctional-detention.html.

## References

[CR1] Akiyama, M. J., Spaulding, A. C., & Rich, J. D. (2020).Flattening the curve for incarcerated populations: COVID-19 in jails and prisons. *The New England Journal of Medicine*. 10.1056/NEJMp2005687 10.1056/NEJMp2005687 PMC7398586 32240582

[CR2] Carson, E. A., & Sabol, W. J. (2016). *Aging of the state prison population, 1993 – 2013*

[CR3] Carson, E. A. (2021). *Prisoners in 2020 – Statistical tables*. https://bjs.ojp.gov/content/pub/pdf/p20st.pdf

[CR4] Carson, E. A. (2021). *Mortality in state and federal prisons, 2001–2019 – Statistical tables*

[CR5] Carson, E. A. (2022). *Prisoners in 2021 - Statistical tables* (Issue December)

[CR6] Carson, E. A., & Nadel, M. (2022). *Impact of COVID-19 on state and federal prisons, March 2020–February 2021*

[CR7] Center for Disease Control and Prevention. (2022). *Intermin list of categories of essential workers mapped to standardized industry codes and titles*. https://www.cdc.gov/vaccines/covid-19/categories-essential-workers.html

[CR8] Charmaz K (2014). Constructing grounded theory.

[CR9] Cloud DH, Ahalt C, Augustine D, Sears D, Williams B (2020). Medical isolation and solitary confinement: Balancing health and humanity in US jails and prisons during COVID-19. Journal of General Internal Medicine.

[CR10] COVID Prison Project. (2023). *National COVID-19 statistics*. https://covidprisonproject.com/

[CR11] Crewe B, Goldsmith A, Halsey M (2022). Power and pain in the modern prison: The society of captives revisited.

[CR12] Fahmy C, Wallace DM, Huebner BM, Frost NA (2018). Incarceration, reentry, and health. Handbook on the Consequences of Sentencing and Punishment Decisions.

[CR13] Fan VY, Jamison DT, Summers LH (2018). Pandemic risk: How large are the expected losses?. Bulletin of the World Health Organization.

[CR14] Ferdik F, Frogge G, Doggett S (2022). “It’s like the zombie apocalypse here:” Correctional officer perspectives on the deleterious effects of the COVID-19 pandemic. Crime and Delinquency.

[CR15] Foss NJ (2020). Behavioral strategy and the COVID-19 disruption. Journal of Management.

[CR16] Freeman RM (1998). The real event model or the organizational covenience model? A national survey of correctional emergency preparedness evaluation methodology. The Prison Journal.

[CR17] Frost NA, Monteiro CE (2020). The interaction of personal and occupational factors in the suicide deaths of correction officers. Justice Quarterly.

[CR18] Gigerenzer, G. (2004). Fast and frugal heuristics: The tools of bounded rationality. In *Blackwell handbook of judgement and decision making* (p. 88).

[CR19] Gigerenzer G, Gaissmaier W (2011). Heuristic decision making. Annual Review of Psychology.

[CR20] Glaser B, Strauss A (1967). The discovery of grounded theory: Strategies for qualitative research.

[CR21] Gofen A (2014). Mind the gap: Dimensions and influence of street-level divergence. Journal of Public Administration Research and Theory.

[CR22] Guest G, Namey E, McKenna K (2017). How many focus groups are enough? Building an evidence base for nonprobability sample sizes. Field Methods.

[CR23] Gutierrez C, Patterson EJ (2021). Risk and implications of COVID-19 among the community supervised population. Criminology and Public Policy.

[CR24] Hallas L, Hatibie A, Majumdar S, Pyarali M, Hale T (2020). Variation in US states’ responses to COVID-19.

[CR25] Hanoch Y (2002). “Neither an angel nor an ant:” Emotion as an aid to bounded rationality. Journal of Economic Psychology.

[CR26] Hawks L, Woolhandler S, McCormick D (2020). COVID-19 in prisons and jails in the United States. JAMA Internal Medicine.

[CR27] Herring, T., & Sharma, M. (2021). *States of emergency: The failure of prison system responses to COVID-19*. https://www.prisonpolicy.org/reports/states_of_emergency.html

[CR28] Hesse-Biber, S. N., & Leavy, P. (2011). Focus group interviews. In *The practice of qualitative research* (pp. 163–192).

[CR29] Holmes S (1995). Passions and constraint: On the theory of liberal democracy.

[CR30] Hummer D (2020). United States Bureau of Prisons’ response to the COVID-19 pandemic. Victims & Offenders.

[CR31] Kaufman BE (1999). Emotional arousal as a source of bounded rationality. Journal of Economic Behavior and Organization.

[CR32] Kinner SA, Young JT, Snow K, Southalan L, Lopez-Acuña D, Ferreira-Borges C, O’Moore É (2020). Prisons and custodial settings are part of a comprehensive response to COVID-19. The Lancet Public Health.

[CR33] Kwan A, Garcia-Grossman I, Sears D, Bertozzi SM, Williams BA (2022). The impact of COVID-19 on the health of incarcerated older adults in California state prisons. Health Affairs (project Hope).

[CR34] Leibowitz AI, Siedner MJ, Tsai AC, Mohraeb AM (2021). Association between prison crowding and COVID-19 incidence rates in Massachusetts prisons, April 2020-January 2021. JAMA Internal Medicine.

[CR35] LeMasters K, Ranapurwala S, Maner M, Nowotny KM, Peterson M, Brinkley-Rubinstein L (2022). COVID-19 community spread and consequences for prison case rates. PLoS ONE.

[CR36] Loftland J, Snow D, Anderson L, Lofland LH (2006). Analyzing social settings: A guide to qualitative observation and analysis.

[CR37] Malloy, G. S. P., Puglisi, L., Brandeau, M. L., Harvey, T. D., & Wang, E. A. (2021). Effectiveness of interventions to reduce COVID-19 transmission in a large urban based analysis jail: A model-based analysis. *BMJ Open, 11*(2). 10.1136/bmjopen-2020-042898 10.1136/bmjopen-2020-042898 PMC7893212 33597139

[CR38] Maner, M., Lemasters, K., Lao, J., Cowell, M., Nowotny, K., Cloud, D., & Brinkley-Rubinstein, L. (2021). COVID-19 in corrections: Quarantine of incarcerated people. *PLos ONE, 16*(10). 10.1371/journal.pone.0257842 10.1371/journal.pone.0257842 PMC8491943 34610015

[CR39] Martin JL, Lichtenstein B, Jenkot RB, Forde DR (2012). “They can take us over any time they want”: Correctional officers’ responses to prison crowding. The Prison Journal.

[CR40] Maycock M (2022). "Covid has caused a dramatic change to prison life": Analysing the impacts of the Covid-19 pandemic on the pains of imprisonment in the Scottish Prison estate. The British Journal of Criminology.

[CR41] Maynard-Moody S, Musheno M (2000). State agent or citizen agent: Two narratives of discretion. Journal of Public Administration Research and Theory.

[CR42] Montoya-Barthelemy A, Lee CD, Cundiff D, Smith E (2020). COVID-19 and the correctional environment: The American prison as a focal point for public health. American Journal of Preventive Medicine.

[CR43] Moseley A, Thomann E (2021). A behavioural model of heuristics and biases in frontline policy implementation. Policy and Politics.

[CR44] Murdoch DJ, King LL (2020). ‘Not feeling like a caged animal:’ Prisoner perceptions of a remote video visitation system. Journal of Crime and Justice.

[CR45] National Conference of State Legislatures. (2022). *COVID-19: Essential workers in the states*. https://www.ncsl.org/research/labor-and-employment/covid-19-essential-workers-in-the-states

[CR46] Nix J, Ivanov S, Pickett JT (2021). What does the public want police to do during pandemics? A national experiment. Criminology and Public Policy.

[CR47] Novisky, M. A., Narvey, C. S., & Semenza, D. C. (2020). Institutional responses to the COVID-19 pandemic in American prisons. *Victims & Offenders*, 1–18. 10.1080/15564886.2020.1825582

[CR48] Novisky MA (2018). Avoiding the runaround: The link between cultural health capital and health management among older prisoners. Criminology.

[CR49] Novisky MA, Narvey CS, Piquero AR (2022). The keepers: Returning citizens’ experiences with prison staff misconduct. Criminal Justice and Behavior.

[CR50] Novisky MA, Nowotny KM, Jackson DB, Testa A, Vaughn MG (2021). Incarceration as a fundamental social cause of health inequalities: Jails, prisons, and vulnerability to COVID-19. The British Journal of Criminology.

[CR51] NVivo (2018). NVivo Data Analysis Software, Version 10.

[CR52] Oladeru, O. T., Tran, N. T., Al-Rousan, T., Williams, B., & Zaller, N. (2020). A call to protect patients, correctional staff and healthcare professionals in jails and prisons during the COVID-19 pandemic. *Health and Justice, 8*(17). 10.1186/s40352-020-00119-1 10.1186/s40352-020-00119-1 PMC7331488 32617825

[CR53] Pedrosa AL, Bitencourt L, Fróes ACF, Cazumbá MLB, Campos RGB, de Brito SBCS, Simões e Silva AC (2020). Emotional, behavioral, and psychological impact of the COVID-19 pandemic. Frontiers in Psychology.

[CR54] Piquero AR (2021). The policy lessons learned from the criminal justice system response to COVID-19. Criminology and Public Policy.

[CR55] Puglisi LB, Brinkley-Rubinstein L, Wang EA (2023). COVID-19 in carceral systems: A review. Annual Review of Criminology.

[CR56] Pyrooz, D. C., Labrecque, R. M., Tostlebe, J. J., & Useem, B. (2020). Views on COVID-19 from inside prison: Perspectives of high-security prisoners. *Justice Evaluation Journal*. 10.1080/24751979.2020.1777578

[CR57] Robbins IP (2008). Lessons from Hurricane Katrina: Prison emergency preparedness as a constitutional imperative. University of Michigan Journal of Law Reform.

[CR58] Schultz, W. J., & Ricciardelli, R. (2022). The floating signifier of “safety:” Correctional officer perspectives on COVID-19 restrictions, legitimacy and prison order. *The British Journal of Criminology*, 1–18. 10.1093/bjc/azac088 10.1093/bjc/azac088 PMC10433504 37600930

[CR59] Simon HA (1953). A behaviour model of rational choice. Quarterly Journal of Economics.

[CR60] Simon HA (1956). Rational choice and the structure of the environment. Psychological Review.

[CR61] Stern, M. F., Piasecki, A. M., Strick, L. B., Rajeshwar, P., Tyagi, E., Dolovich, S., Patel, P. R., Fukunaga, R., & Furukawa, N. W. (2021). *Willingness to receive a COVID-19 vaccination among incarcerated or detained persons in correctional and detention facilities — Four states, September-December 2020* (Vol. 70, Issue 13). 10.15585/mmwr.mm7013a3 PMC8022882 33793457

[CR62] Strauss AL (1987). Qualitative analysis for social scientists.

[CR63] Sykes GM (1958). The society of captives: A study of a maximum security prison.

[CR64] Tummers LLG, Bekkers V, Vink E, Musheno M (2015). Coping during public service delivery: A conceptualization and systematic review of the literature. Journal of Public Administration Research and Theory.

[CR65] U.S. Bureau of Labor Statistics. (2021). *Occupational employment and wages, May 2021: 33–3012 correctional officers and jailers*. https://www.bls.gov/oes/current/oes333012.htm

[CR66] Useem B, Kimball P (1991). States of siege: US prison riots, 1971–1986.

[CR67] Wasti SP, Simkhada P, van Teijlingen E, Sathian B, Banerjee I (2022). The growing importance of mixed-methods research in health. Nepal Journal of Epidemiology.

[CR68] Wildeman C, Wang EA (2017). Mass incarceration, public health, and widening inequality in the USA. The Lancet.

[CR69] Williams BA, Ahalt C, Cloud D, Augustine D, Rorvig L, Sears D (2020). Correctional facilities in the shadow of COVID-19: Unique challenges and proposed solutions.

[CR70] Williams B, DiTomas M, Pachynski A (2021). The growing geriatric prison population: A dire public health consequence of mass incarceration. Journal of the American Geriatrics Society.

[CR71] Worley, R. M., Lambert, E. G., & Worley, V. B. (2022).Can’t shake the prison guard blues: Examining the effects of work stress, job satisfaction, boundary violations, and the mistreatment of inmates on the depressive symptomatology of correctional officers. *Criminal Justice Review*. 10.1177/07340168221123229

