# Correction: “The COVID‑19 pandemic and operational challenges, impacts, and lessons learned: a multi‑methods study of U.S. prison systems”

**DOI:** 10.1186/s40352-024-00278-5

**Published:** 2024-05-25

**Authors:** Meghan A. Novisky, Jennifer Tostlebe, David Pyrooz, Jose Antonio Sanchez

**Affiliations:** 1 Department of Criminology and Sociology, Cleveland State University, 2121 Euclid Avenue, UR 205, Cleveland, OH 44115 USA; 2 School of Criminology and Criminal Justice, University of Nebraska Omaha, Omaha, NE USA; 3 Department of Sociology, University of Colorado Boulder, Boulder, CO USA

**Correction: Health Justice 11, 51 (2023)**

**https://doi.org/10.1186/s40352-023-00253-6**

Following the publication of the original article (Novisky et al., 2023), the authors amended the Acknowledgements section to acknowledge the authors partnership with the Center for Justice Research and Innovation.

The statement in the Acknowledgements has been amended from:

“The authors would like to thank the NIC and all respondents who consented to participate in the research.”

To:

“This research was a result of a collaboration between the authors and CNA’s Center for Justice Research and Innovation. The authors would like to thank CNA for their partnership, as well as the NIC and all respondents who consented to participate in the research.”

The original article (Novisky et al., 2023) has been updated.
